# Effects of low-intensity exercise on spontaneously developed knee osteoarthritis in male senescence-accelerated mouse prone 8

**DOI:** 10.1186/s13075-023-03162-z

**Published:** 2023-09-14

**Authors:** Kosuke Norimatsu, Kazuki Nakanishi, Toshiro Ijuin, Shotaro Otsuka, Seiya Takada, Akira Tani, Ryoma Matsuzaki, Teruki Matsuoka, Harutoshi Sakakima

**Affiliations:** 1 Department of Physical Therapy, School of Health Sciences, Faculty of Medicine, Kagoshima University, 8-35-1, Sakuragaoka, Kagoshima, 890-8544 Japan; 2 Department of Orthopaedic Surgery, Kagoshima University, Kagoshima, Japan; 3 Department of Systems Biology in Thromboregulation, Kagoshima University Graduate School of Medical and Dental Science, Kagoshima, Japan

**Keywords:** Aging, Osteoarthritis, Exercise, Rehabilitation, Mouse model, Synovial inflammation, M1 macrophage

## Abstract

**Background:**

Osteoarthritis (OA) is a degenerative joint disease associated with aging, which often leads to joint stiffness and disability. Exercise is one of the most important non-pharmacological treatments and is prescribed as an indispensable treatment for OA. However, whether physical exercise is beneficial for preventing the progression of OA symptoms with age is poorly understood. We investigated the effects of exercise on spontaneously developed knee OA using male senescence-accelerated mouse prone 8 (SAMP8).

**Methods:**

To examine age-related changes in the knee joints of SAMP8, knee articular cartilage changes, synovitis, knee joint flexion and extension angles, swelling, walking ability, and quadriceps muscle atrophy were analyzed at 3, 5, 7, and 9 months. SAMP8 were required to run at a speed of 10 m/min for 15 min/day from 7 to 9 months of age. The knee joint pathologies and symptoms of exercising and non-exercising mice were compared by histological, immunohistochemical, and morphometrical analyses.

**Results:**

The mice presented with various histological changes, including cartilage destruction, osteocyte formation, synovitis, declined joint angles, and swelling. Notably, medial and posterior cartilage destruction was more severe than that of the lateral and anterior cartilage. Knee joint angles were significantly correlated with the histological scores (modified Mankin and OARSI, osteophyte formation and synovial lining cell layer). Exercise did not attenuate cartilage degeneration in the medial and posterior tibial plateau, although the articular cartilage of the anterior and lateral tibial plateau and its histological scores was remained and significantly improved, respectively, by exercise. Exercise suppressed the age-related decline of collagen type II-positive areas in the remaining articular cartilage and improved the OA symptoms. Exercise reduced the expression of monocyte chemoattractant protein (MCP)-1 and tumor necrosis factor (TNF)-α positive macrophages in the synovium.

**Conclusion:**

This study revealed that SAMP8 developed spontaneous knee OA with age, which resembled the disease symptoms in humans. Low-intensity exercise temporarily alleviated degeneration of the remaining cartilage, synovitis, and age-related decreases in knee flexion angle, stride length, and muscle atrophy in SAMP8. However, exercise during OA progression with age may cause mechanical stress that could be both beneficial and detrimental to joint health.

**Supplementary Information:**

The online version contains supplementary material available at 10.1186/s13075-023-03162-z.

## Introduction

Osteoarthritis (OA) is one of the most common arthritic diseases prevalent in older people, causing severe disability [1]. OA is a degenerative joint disease characterized by progressive cartilage and subchondral bone degeneration, osteophyte formation, and synovial inflammation, which lead to joint stiffness and disability [2, 3]. The complex involvement of several factors such as aging, obesity, and severe mechanical stress is associated with the development of OA.

Medications and non-pharmacological treatments are recommended for OA [4, 5]. However, effective drugs to treat OA are lacking and therapies are mainly aimed at managing OA symptoms such as joint stiffness and pain. Currently, exercise is one of the most important non-pharmacological treatments and indispensable in treating OA. However, in a recent review article, exercises were shown to be both beneficial and detrimental to articular cartilage health in humans [3]. In animal studies, daily moderate exercise may have positive effects on cartilage matrix composition in healthy animals and post-traumatic OA, unlike high-intensity exercise [6, 7]. In addition, moderate treadmill exercise for 3 days/week inhibited cartilage degeneration and synovitis in mice with anterior cruciate ligament transection (ACL-T) as an OA model [8]. However, excessive eccentric muscular exercise, such as downhill training, increases the risk of OA by inducing a chronic pro-inflammatory state in mice [9]. Overall, low and moderate exercise regimens may be beneficial in preventing OA progression. However, the role of physical exercise in preventing OA progression with aging has been debated, as its effects are not well understood.

Although aging is an important contributing factor to the development of OA, several experimental OA models in young animals have been used to address the molecular pathogenesis of OA and effects of exercise interventions, including surgical OA models, such as ACL-T [10], destabilization of the medial meniscus [11], and meniscectomy [12]. Other models use toxins or small chemical compounds that induce chondrocyte apoptosis or joint inflammation, such as monosodium iodoacetate [13] or carrageenan [14]. However, these models seemed to involve the pathogenesis of secondary (traumatic) OA, which only accounts for approximately 12% of the overall prevalence of symptomatic OA [15].

Senescence-accelerated mouse (SAM) was derived from an AKR/J breeding colony, a group of related inbred strains expressing the phenotypes of age-associated diseases [16]. Selective breeding has led to the development of several sublines of SAM prone (SAMP) and SAM resistant (SAMR) [16]. For example, P strains exhibit senile amyloidosis (P1, P2, P7, and P11), degenerative arthrosis of the temporomandibular joint (P3), senile osteoporosis (P6), thymoma (P7), brain atrophy-related deficits in learning and memory (P8 and P10), and cataracts (P9) [16, 17]. SAMP share similar characteristics to elderly humans, such as a reduced lifespan, lordosis, hair loss, decreased physical activity, and cognitive impairment [17]. SAMP8 includes phenotypes with mutations in the mitochondrial genome that cause a progressive increase in systemic oxidative stress and premature aging [18, 19]. However, the genes responsible for accelerated aging commonly seen in this strain are not completely understood. Recently, several studies observed early histological and pathological cartilage and subchondral bone changes in the knee joints of SAMP8 and reported that these mice act as spontaneous OA models [19–21]. These studies revealed bone changes at 8 months of age and cartilage damage, osteophyte formation, and synovitis in 11-month-old SAMP8 [19, 20]. However, it is unclear whether SAMP8 presented age-related OA symptoms that are consistent with human OA, including reduced joint mobility, swelling, walking difficulty, and muscle atrophy.

Therefore, this study investigated not only age-related knee OA but also knee-joint motion, swelling, walking ability, and quadriceps muscle atrophy in SAMP8. Furthermore, this study examined whether exercise could alleviate knee OA pathology and the symptoms associated with aging in these mice.

## Materials and methods

### Animals

A total of 52 1-month-old male SAMP8 (23.2 ± 1.7 g, mean ± SD) and a 13 age-matched SAMR1 (22.1 ± 0.8 g) were obtained from Japan SLC, Inc. animal suppliers (Hamamatsu, Japan). Two or three mice were housed in each temperature-controlled cage (23.0 °C ± 1.0 °C) with a 12-h light/dark cycle with unlimited access to food and water. SAMR1 were used as controls for SAMP8. The animals were randomly assigned to experimental groups.

### Experimental design

To clarify the symptomatic progression of spontaneous OA, we investigated age-related histopathological and immunohistochemical changes of the knee joint, knee joint morphometry (including knee joint angle and width), walking ability, quadriceps muscle cross-sectional area (CSA), and knee joint radiographs in SAMP8 aged 3, 5, 7, and 9 months (*n* = 3–6 per month). Subsequently, we examined whether low-intensity treadmill exercise regimens (described below) could alleviate the spontaneously developed knee OA symptoms in SAMP8. Knee joints of SAMR1 were observed at 7 and 9 months of age (*n* = 6–7 per month). In this study, we used the data of 7- and 9-month-old SAMR1 as controls because there were no significant differences in their knee joint histopathological changes, motor functions, morphometry, and quadriceps muscle CSA between these months. Furthermore, we used the data from 9-month-old SAMR1 as controls for immunohistochemical analyses. We minimized potential confounders, such as the order of treatments and measurements, or animal/cage location, to a possible extent. All experimental procedures were performed in compliance with the protocol guidelines established by the Institute of Laboratory Animal Sciences of Kagoshima University and were approved by the ethical committee of our institution (No. M20001).

### Treadmill exercise protocols

Treadmill exercises were performed using a motor-driven treadmill (MK-680; Muromachi Kikai Co., Ltd, Japan). All mice were subjected to treadmill running at a speed of 10 m/min for 15 min/day for 3 days (familiarization). After familiarization, the mice were required to run at a speed of 10 m/min for 15 min/day, at an inclination angle of 0°, for 10 weeks (from 7 to 9 months of age) (Ex group; *n* = 10). Mice of the No-Ex group (*n* = 6) and 9-month-old SAMP8 were similar, because these mice were not exercised from 7 to 9 months of age. Generally, treadmill running at a speed of 10–12 m/min is a low-intensity exercise regimen for mice. Exercise was performed at room temperature during the day (10:00–16:00). Body mass was periodically measured to monitor the stress induced by treadmill exercise and explain potential mass differences between the Ex and No-Ex groups.

### Radiographic evaluation and macroscopic observation of the knee joint

To examine age-related changes in the knee joints of SAMP8 and SAMR1 aged 3, 5, 7, and 9 months (*n* = 3 per age group), we performed radiographic examinations using portable X-ray equipment (KX-60, Asahi Roentgen Ind. Co., Ltd, Kyoto, Japan). X-ray radiography was performed at 60 kV and 10 mA for 0.2 s from a height of 18 cm under 2.0% isoflurane-induced anesthesia (Pfizer Inc., Tokyo, Japan) using the MK-A110 small animal anesthetizer (Muromachi Kikai, Tokyo, Japan).

To examine changes in the joint capsule and cartilage surface, we performed macroscopic observations of the tibiofemoral joint in 8- or 9-month-old SAMP8 (*n* = 5) and control mice (*n* = 4). Both tibiofemoral joints were carefully removed with a joint capsule, and the tibia and femoral condyles were subsequently dissected separately without damaging the cartilage surface. Macroscopic images were obtained using a microscopic monitor camera (MIC-142, AS ONE Corp., Osaka, Japan).

### Walking analyses

To analyze age-related locomotory changes, the walking speed and number of steps were measured using a beam walking task (30 × 1.0 [1xw]) at the ages of 3, 5, 7, and 9 months, and Ex, No-Ex, and control (*n* = 6 per age group). A digital video of the crossing of the beam was captured using a video camera (HC-V520M, Panasonic Corp., Ltd, Tokyo, Japan) and analyzed by two individuals on a computer. The time to traverse the beam was recorded in two or three trials per individual. The best value for traversing the beam was used for the analysis, and the walking speed and stride length of each individual were calculated for that particular recording.

### Histological assessment

Six SAMP8 per age group were euthanized at 3-, 5-, 7-, and 9-month-old. The Ex group also underwent histological analysis. SAMR1 were euthanized at 7- and 9-month-old (*n* = 6 per age group). Mice were deeply anesthetized with sodium pentobarbital and perfused through the heart with heparinized physiological saline. After both the thigh and calf muscles were removed, the knee joints were harvested and immersion-fixed in 4% paraformaldehyde in 0.1 M phosphate buffer (pH 7.4) overnight at 4 °C. After decalcification with KalkitoxTM (FUJIFILM Wako Pure Chemical Corp, Japan) for 3–5 days at 4 °C, the samples were neutralized with 5% sodium sulfate solution overnight at room temperature and washed in distilled water for 1 h. The right knee joint was embedded in paraffin to obtain coronal sections that contained the central weight-bearing regions of the medial and lateral tibiofemoral joints. The left knee joint was embedded in paraffin to obtain sagittal sections of the medial mid-condylar level. The coronal and sagittal Sects. (5 μm) were stained with hematoxylin and eosin (H&E) and safranin-O fast green to evaluate articular cartilage, osteophyte formation, and synovium in each knee joint.

Articular cartilage in the tibial plateau was evaluated using the modified Mankin score (score: 0–15) and the Osteoarthritis Research Society International (OARSI) osteoarthritis pathology assessment system (score: 0–24), which is a histological scoring system for articular cartilage quality [22–24] using sagittal sections. We evaluated the remaining articular cartilage because considerable loss of the cartilage layer was observed in the SAMP8. In addition, we evaluated the percentage of medial and posterior cartilage destruction in SAMP8 and SAMR1 with age (*n* = 6–10 per group). Osteophyte formation was evaluated using a 0–3 histological grading score (0, none; 1, formation of cartilage-like tissue; 2, increase in cartilaginous matrix; and 3, endochondral ossification) in the mouse model [12]. Osteophyte formation scores were measured in the medial, lateral, and posterior regions of the tibial plateau using the coronal and sagittal sections, and the average score was analyzed. Synovial thickening was evaluated using a 0–3 scoring system (0, no synovial thickening; 1, lining of two cell layers; 2, several extra cell layers; and 3, clear inflammation with infiltrate or exudate) [25]. Synovial thickening scores were measured in the medial, lateral, and posterior regions using the coronal and sagittal sections, and the average score was used for the analysis. The quantitative analysis of the scoring system was performed by two individuals.

The CSA of the right quadriceps muscle was measured at 3, 5, 7, and 9 months SAMP8 and the SAMR1 control (*n* = 6 per age group). The quadriceps muscle was mounted vertically on a cork plate in tragacanth gum jelly to obtain a cross-section at approximately 5 mm from the upper part of the knee joint, which was quickly frozen in isopentane chilled with liquid nitrogen, and stored at – 80 °C for subsequent analysis. Transverse Sects. (10 μm) were cut with a cryostat microtome at – 20 °C and stained with H&E to measure muscle CSA. H&E sections were imaged at × 10 magnification, and the muscle CSA was quantitatively measured by two individuals. A random sample of more than 100–120 fibers from each muscle was measured using ImageJ software version 1.53 (NIH, USA).

### Morphometric assessments of knee joints

Both the knee joint flexion and extension angles were measured at 3 (*n* = 3), 5 (*n* = 3), 7 (*n* = 5), and 9 months (*n* = 6) of age in SAMP8, and the control (*n* = 6–7). To examine the effects of the joint structure itself, the thigh (quadriceps femoris and hamstring muscles) and calf (gastrocnemius, soleus, and plantar) muscles were removed at the knee joint and used to measure the knee joint angles. The mice were placed in a neutral lateral recumbent position, and the flexion and extension angles in the maximally extended position were measured as 0°. A force of 0.03 N of knee flexion or extension moment was applied to the distal portion of the hindlimb using a digital strain amplifier (WDS-180A, Kyowa Electronic Instrument Co., Ltd, Tokyo, Japan). The angle between the femur and fibula was photographed and printed on paper, and the acute angle of the femur and fibula formed by the three points of the femoral greater trochanter, center of the patella, and lateral malleolus were measured as knee flexion and extension angles using a protractor (Fig. S1A-D). Knee joint angles on both sides were used in the analysis.

To examine swelling in the knee joint, the width of the knee joint was measured in mice aged 3 (*n* = 3), 5 (*n* = 3), 7 (*n* = 5), and 9 months (*n* = 6) and in control mice (*n* = 4–6). Hind limbs on both sides where the thigh and calf muscles were removed were used to measure the width of the knee joint, and the maximal width in the knee center was measured (Fig. S1E, F). Quantitative analyses were performed using ImageJ software version 1.53 (NIH, USA), and the average of the widths measured by two individuals were used for the statistical analysis.

### Immunohistochemical analysis

Immunostaining was performed to assess the activity of factors involved in cartilage matrix degradation and synovitis. The coronal or sagittal sections were stained with the following antibodies: rabbit anti-collagen type II antibody (marker of the development and maturation process of chondrocytes) (Abcam plc, Cambridge, UK; ab34712), rabbit anti-matrix metalloproteinase (MMP)-13 antibody (marker of proteolytic enzyme) (Abcam plc; ab39012), rabbit anti-monocyte chemoattractant protein (MCP)-1 antibody (marker of the chemokine that recruits circulating inflammatory cells) (Abcam plc; ab25124), and rabbit anti-tumor necrosis factor (TNF)-α antibody (marker of pro-inflammatory macrophages) (Abcam plc; ab6671). Following deparaffinization and rehydration, the endogenous peroxidase was blocked with methanol containing 3.0% hydrogen peroxide for 10 min. The sections were then rinsed three times (5 min each) in phosphate-buffered saline (PBS, pH 7.6) and blocked with 10% skimmed milk in PBS for 20 min. All sections were individually incubated at 4 °C overnight with the following antibodies: rabbit anti-collagen type II (1:50), rabbit anti-MMP-13 (1:50), rabbit anti- MCP-1 (1:100), and rabbit anti-TNF-α (1:50). The sections were then washed in PBS three times for 5 min each and incubated for 60 min with goat anti-rabbit IgG conjugated to a peroxidase-labeled dextran polymer (EnVision; Dako, CA, USA). Finally, the sections were rinsed with PBS and their immunoreactivity was visualized using diaminobenzidine staining.

**Fig. 1:**
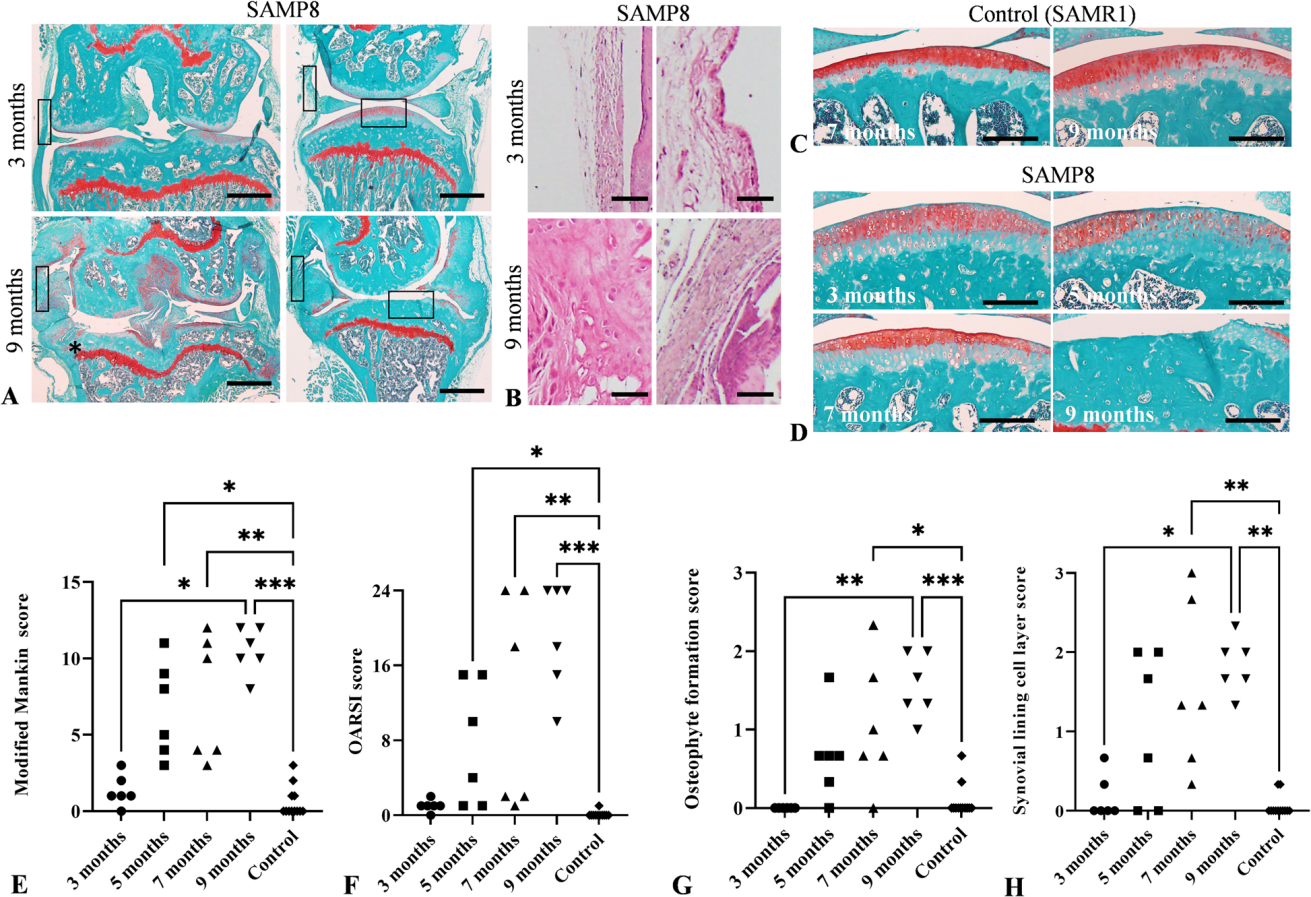
OA development, indicated as cartilage changes, osteophyte formation, and changes in the synovial lining cell layer with age. **A** Comparison of representative knee joints in 3- and 9-month-old SAMP8 using safranin-O staining. The 9-month-old mice had severe cartilage degradation extending to the subchondral bone and osteophyte formation (*) in the medial cartilage edge compared with the 3-month-olds. **B** H&E staining of synovium shown in the left and right vertical rectangle in **A**, respectively. The synovial lining cell layer in 9-month-old mice increased compared with that in 3-month-old mice. **C**, **D** Safranin-O staining of the representative cartridge changes in the control and SAMP8. Histological observations in the tibial plateau were performed, shown in the right horizontal rectangle in **A**. SAMP8 had irregular and reduced safranin-O staining on the cartilage surface in the tibial plateau that gradually increased with age. **E** Changes in the modified Mankin score. **F** Changes in the OARSI score. **G** Changes in the osteophyte formation scores. **H** Changes in the synovial lining cell layer score. The data from 7- and 9-month-old SAMR1 were used as a control. *p < 0.05, **p < 0.01. Scale bars = 500 μm (**A**), 50 μm (**B**), 200 μm (**C**, **D**). (*n* = 6–7 per age group)

Immunostained sections obtained at 3, 5, 7, and 9-month-old SAMP8, the control (*n* = 6 per age group), and the Ex group (*n* = 9–10) were used for semi-quantitative analysis. Articular cartilage in collagen type II and MMP-13 immunostained sagittal sections was imaged at × 20 magnification using a microscope and camera. The number of collagen type II and MMP-13 positive chondrocytes per unit area (104 μm^2^) was quantitatively measured in the articular cartilage of the tibial plateau (Fig. 1A, horizontal rectangle). MCP-1- and TNF-α-immunostained sections were imaged at × 10 magnification using the same system described above. The proportion of MCP-1-and TNF-α-positive cell areas were measured in the medial, lateral, and posterior synovium regions using coronal and sagittal sections, and the average percentages of immunolabeled area per total area were used for analysis. Semi-quantitative analysis was performed in the same regions as the histological examination. Rectangular areas with a width of approximately 500 μm in the cartilage and synovium were used for semi-quantitative analyses (Fig. 1A, vertical rectangle). All semi-quantitative analyses were performed using ImageJ software version 1.53 (NIH, USA), and quantitative analysis of each immunolabeled area was performed by two individuals. The measurement threshold for representing immunolabeled cells in the ImageJ software was almost fixed for each animal.

### Statistical analysis

Statistical analyses were performed using both parametric and non-parametric tests following the Shapiro–Wilk test. Either the Mann–Whitney *U* test or independent Student’s *t*-test was used for between-group analyses. The percentage of immunostained areas was analyzed using either one-way analysis of variance (ANOVA) or the Kruskal–Wallis test, followed by Bonferroni's post-hoc tests for multiple comparisons when *p* < 0.05. All data were analyzed using GraphPad Prism 9.3.1 (GraphPad software, LLC, San Diego, CA, USA). Data were statistically processed after outlier removal using the ROUT method in GraphPad Prism software. Spearman’s rank correlation was used to correlate the knee joint angles and histological assessments using SPSS Statistics version 28 (IBM Corp., Armonk, NY, USA). Data are presented as means and 95% confidence intervals (mean ± 95% CI). Statistical significance was set at *p* < 0.05.

## Results

### Histological and immunohistochemical changes in the articular cartilage, osteophyte formation, and synovium

Histological examination of the tibial plateau was performed using H&E and safranin-O staining to determine the OA development time course, including changes in cartilage, osteophyte formation, and synovial lining cell layer (Fig. 1). Three-month-old SAMP8 had a slightly irregular surface and reduced safranin-O staining, whereas 9-month-old mice had severe cartilage degradation extending to the subchondral bone. The modified Mankin and OARSI scores gradually increased with age, and the modified Mankin score increased significantly in 9-month-old mice compared to 3-month-old mice (Fig. 1A–F; *p* = 0.001). Notably, histological observations showed that medial and posterior cartilage destruction was more severe than that of the lateral and anterior cartilage in aged SAMP8 (Table 1).

**Table 1:** Percentage of medial and posterior articular cartilage degeneration in the tibial plateau in SAM

	% of medial cartilage surface degeneration^a^	% of posterior cartilage surface degeneration
SAMP8		
3 months	0.0% (0/6)	0.0% (0/6)
5 months	67.0% (4/6)	0.0% (0/6)
7 months	71.4% (5/7)	12.5% (1/8)
9 months	75.0% (6/8)	50.0% (4/8)
SAMR1		
7 months	0.0% (0/6)	16.6% (1/6)
9 months	0.0% (0/8)	0.0% (0/8)
Treadmill		
No-Ex	75.0% (6/8)	50.0% (4/8)
Ex	60.0% (6/10)	70.0% (7/10)

^a^% of articular cartilage with 3-point difference between the medial and lateral plateau in the modified Mankin score. Knee joint on both sides were observed in each group (number of positive mice/numbers of observed mice). Animals of No-Ex group were equal to the 9-month-old SAMP8

Therefore, we analyzed macroscopic observations of the joint capsule and cartilage surface at 8 or 9 months (Fig. S2). The medial joint capsule was thicker than the lateral part (Fig. S2A). The cartilage surfaces of both the femoral condyle and tibial plateau in SAMP8 were irregular compared with those in the control mice. SAMP8 showed degenerative changes in the posterior articular surface of the medial tibial plateau compared to control mice (Fig. S2B). The mean osteophyte formation scores in 9-month-old mice were significantly higher than those of 3-month-old mice (*p* = 0.001), and this score gradually increased with age (Fig. 1G). Similarly, the mean synovial lining cell layer score was significantly higher in 9-month-old mice than that in 3-month-old mice (*p* = 0.029), with this score also increasing gradually with age (Fig. 1H).

Immunohistochemical examinations were then performed cartilage changes and synovial inflammation proceeds (Fig. S3). The immunoreactivity of collagen type II- and MMP-13-positive chondrocytes gradually decreased from 5 months of age (Fig. S3B, C). The number of MMP13-positive chondrocytes was decreased in 9-month-old mice than that in control mice (Fig. S3 C). The immunoreactivity of MCP-1-positive cells, including a chemokine marker that recruits circulating inflammatory cells in the synovium, gradually increased with age (Fig. S3D). The percentage of TNF-α-positive cells increased significantly in 9-month-old compared to 7-month-old mice (Fig. S3E, *p* = 0.018).

### Assessments of knee joint motion, swilling, stride length, and quadriceps muscle atrophy

**Fig. 2:**
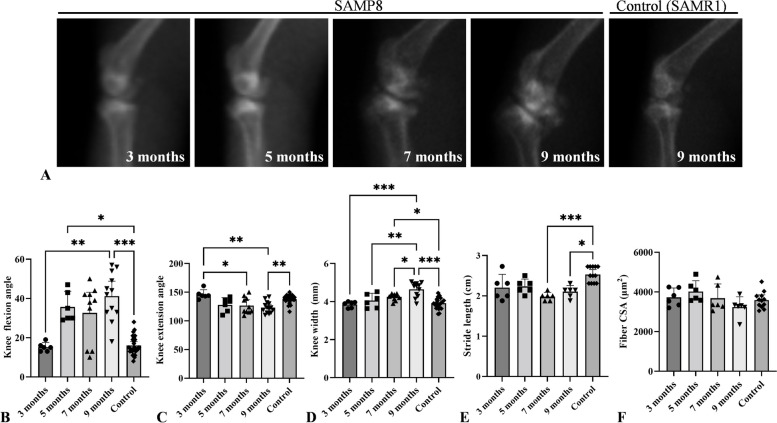
Radiological analysis and morphometrical and walking assessments with age. **A** Time course of radiological analysis of knee joints. In 9-month-old mice, advanced osteophyte formation or joint destruction was recognized at the posterior part of the joint compared with that in the 3-month-old and control mice. **B** Changes in knee joint flexion angle. **C** Changes in knee joint extension angle. **D** Changes in width of knee joint. **E** Changes in stride length. **F** changes in the quadriceps muscle fiber cross sectional area (CSA). Data are expressed as the means ± 95% CI. *p < 0.05, **p < 0.01, ***p < 0.001. (*n* = 3–7 per age group)

We performed radiological analysis of the knee joints (Fig. 2A). No apparent osteophyte formation or joint destruction was observed in 3-month-old SAMP8 and control mice. However, osteophyte formation or joint destruction in the posterior region was recognized in 9-month-old mice, suggesting that these changes progressed with age.

We then analyzed age-related changes in knee joint angles, swelling, stride length, and quadriceps muscle fiber CSA (Fig. 2B–F). Knee flexion and extension angles deteriorated with age. Notably, the deterioration was more significant in 9-month-old mice than that in 3-month-old and control mice (Fig. 2B, C, p = 0.008 and 0.001, respectively). The width of the knee joint increased with age and was significantly larger in 9-month-old mice than in the younger and control mice (Fig. 2D). The mean walking speed in 9-month-old mice (0.16 m/s) was lower than that in control mice (0.22 m/s), and the stride length in 7- or 9-month-old mice decreased significantly compared to that in control mice (Fig. 2E, p = 0.001 and 0.035, respectively). The quadriceps muscle fiber CSA decreased with age, but differences between age groups were not significant (Fig. 2F).

In addition, we investigated the relationship between the knee joint angles and histological scores. Knee flexion and extension angles were significantly correlated with the modified Mankin and OARSI, osteophyte formation, and synovial lining cell layer scores (Table 2), suggesting that histological changes in the knee joint may be associated with joint motion loss.

**Table 2:** Correlation coefficients between the knee joint angles and histological scores in SAMP8

	Knee flexion angle	Knee extension angle
Modified Mankin score	*r* = 0.72 (*p* = 0.001)	*r* = − 0.48 (*p* = 0.009)
OARSI score	*r* = 0.71 (*p* = 0.001)	*r* = − 0.48 (*p* = 0.006)
Osteophyte formation score	*r* = 0.71 (*p* = 0.001)	*r* = − 0.48 (*p* = 0.01)
Synovial lining cell layer score	*r* = 0.69 (*p* = 0.001)	*r* = − 0.41 (*p* = 0.029)

Data is shown as correlation coefficients (*p* value). *n* = 28

### The effects of low-intensity exercise on knee articular cartilage

**Fig. 3:**
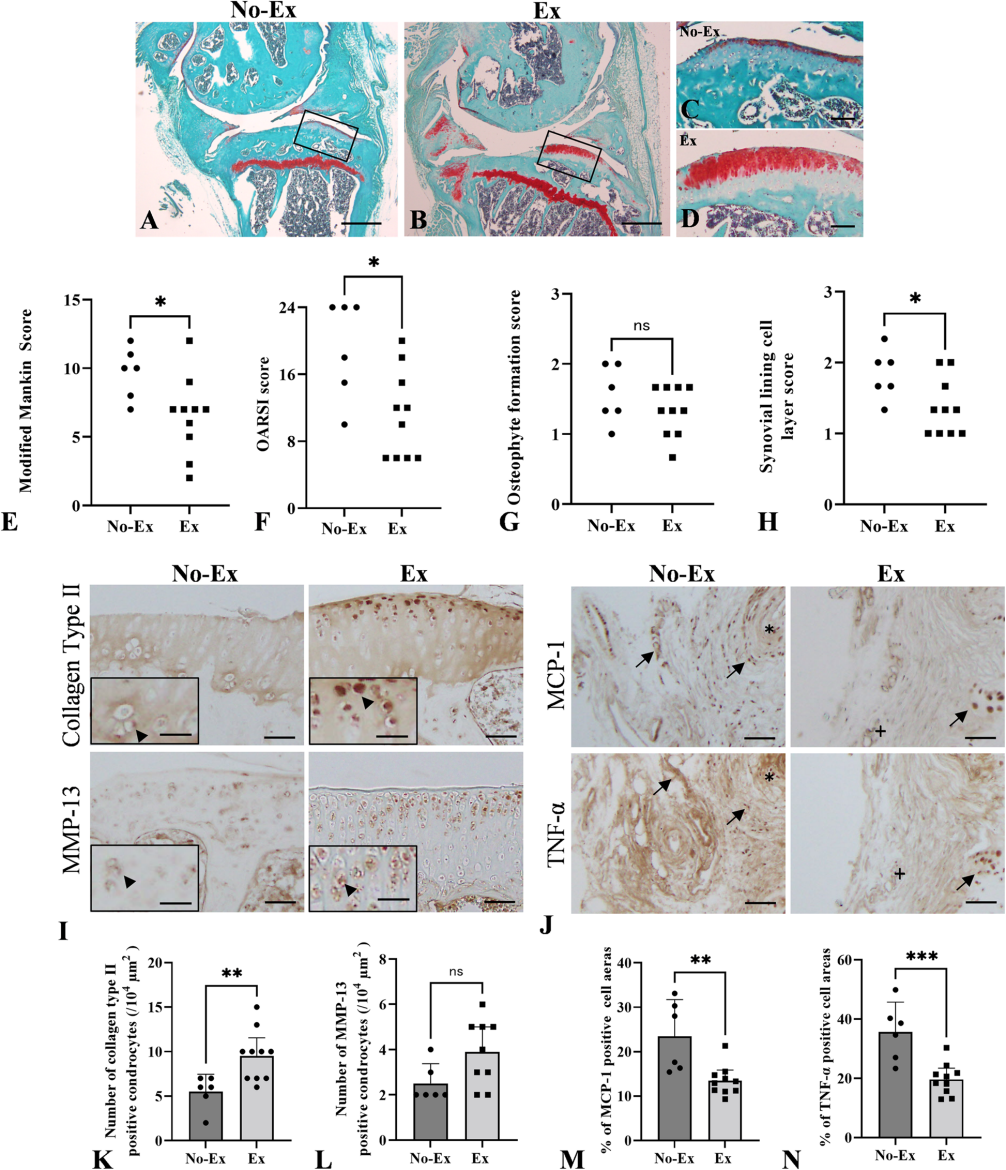
Comparison of histological and immunohistochemical analyses on knee OA between the No-Ex and Ex groups. **A**, **B** Safranin-O staining on the cartilage surface in the tibial plateau and synovium in the No-Ex and Ex groups. **C** High magnification of the rectangular area in **A** (No-Ex). **D** High magnification of the rectangular area of **B** (Ex). The remaining articular cartridge was observed in the anterior tibial plateau in the Ex group. However, posterior cartilage in the tibial plateau was degenerated in both groups. **E** The modified Mankin score. **F** The OARSI score. **G** The osteophyte formation score. **H** The synovial lining cell layer score. **I** Immunoreactivities of collagen type II- and MMP-13 positive chondrocytes in the remaining cartilage of the tibial plateau. High magnification panels show immune-positive cells (black arrow head) for collagen type II- and MMP-13-positive chondrocytes. **J** MCP-1 and TNF-α positive cells (arrow) in the posterior synovium (semi-serial section of the No-Ex and Ex groups, corresponding to * and + , respectively). **K** The number of collagen type II-positive chondrocytes. **L** The number of MMP-13 positive-chondrocytes. **M** The percentage of MCP-1-positive cell areas in the synovium. **N** The percentage of TNF-α-positive cell areas in the synovium. Animals in the No-Ex group were equivalent to 9-month-old SAMP8. Data are expressed as the means ± 95% CI. *p < 0.05, **p < 0.01, ***p < 0.001. Scale bars = 500 μm (**A**, **B**), 100 μm (**C**, **D**), 50 μm (**I**, **J**), and 25 μm (high magnification panels of **I**) (*n* = 6–10 for both groups)

Low-intensity treadmill exercise was performed in mice to investigate whether it alleviated age-related articular cartilage degradation and synovitis. Histological observations showed cartilage destruction, osteophyte formation, and synovial thickening in the No-Ex and Ex groups (Fig. 3A–D). Similar articular cartilage degradation in the medial tibial plateau was observed in the Ex and No-Ex groups (Table 1). Furthermore, degeneration of posterior cartilage in the tibial plateau was greater in the Ex than that in the No-Ex group (Table 1). Furthermore, thickness of articular cartilage in the anterior tibial plateau decreased in the No-Ex group (Fig. 3C). However, the Ex group retained the thickness of articular cartilage in the anterior tibial plateau stained with safranin-O compared to the No-Ex group (Fig. 3D). The modified Mankin score of the remaining cartilage in the anterior tibial plateau, OARSI, and the synovial lining cell layer scores improved significantly in the Ex group compared to the No-Ex group (Fig. 3E, F, H p = 0.03, 0.021 and 0.041, respectively). No significant difference was observed in the osteophyte formation score between the Ex and No-Ex groups (Fig. 3G).

Immunohistochemical analyses of the remaining cartilage in the anterior tibial plateau showed that the number of collagen type II-positive chondrocytes was significantly higher in the Ex than that in the No-Ex group (Fig. 3I, K, p = 0.009). The number of MMP-13-positive chondrocytes increased in the Ex group, but there was no significant difference between the groups (Fig. 3J, L). The percentage of MCP-1- and TNF-α-positive cells was significantly lower in the Ex than that in the No-Ex group, suggesting that exercise ameliorated synovial inflammation (Fig. 3J, M, N, p = 0.003 and 0.001, respectively).

### The effects of low-intensity exercise on knee joint motion, swelling, stride length, and quadriceps muscle atrophy

**Fig. 4:**
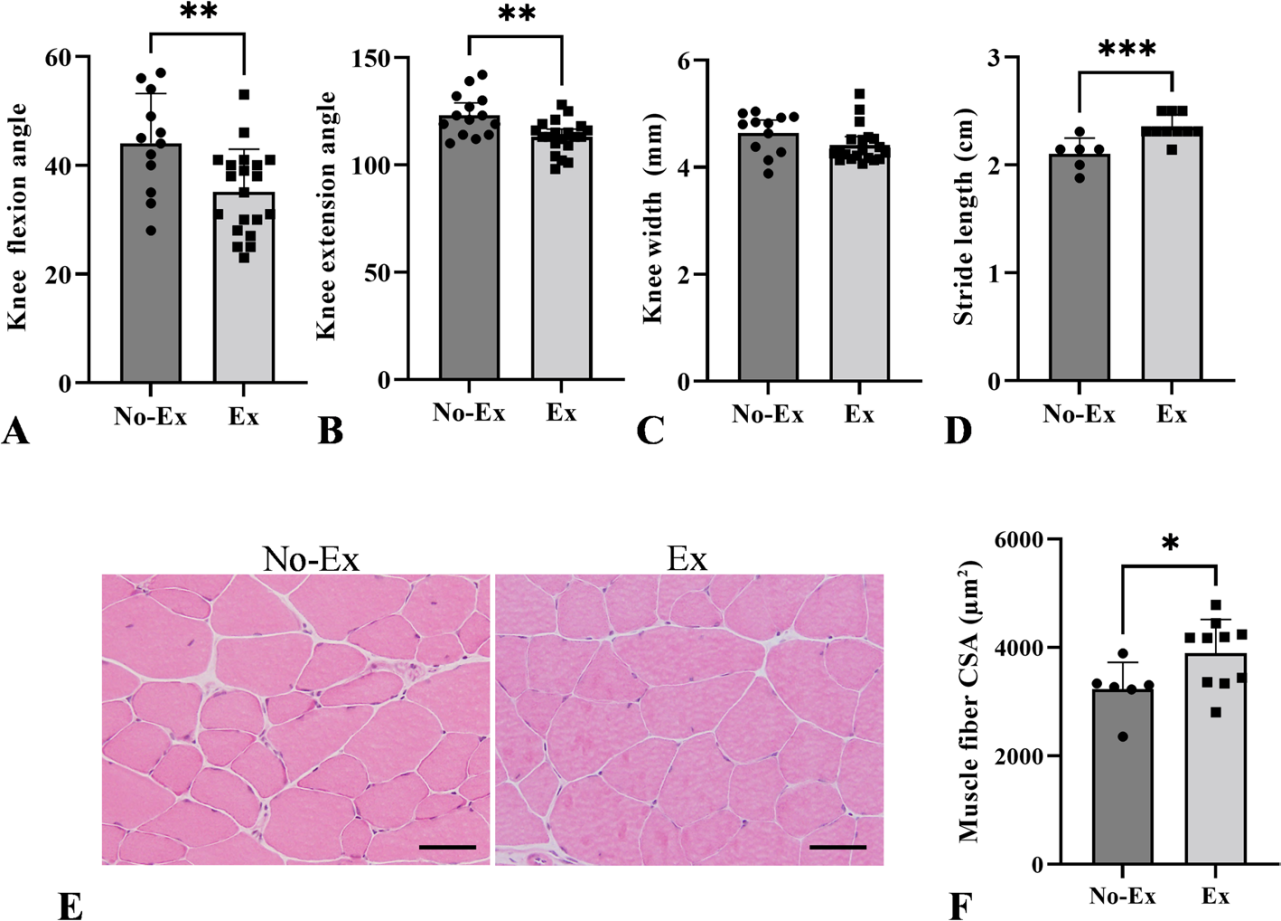
Comparison of morphometric and walking assessments between the No-Ex and Ex groups. **A** Knee joint flexion angle. **B** Knee joint extension angle. **C** Width of knee joint. **D** Stride length. **E**, **F** Quadriceps muscle fiber cross-sectional area. Data are expressed as the means ± 95% CI. *p < 0.05, **p < 0.01, ***p < 0.001. Scale bar = 50 μm (**F**) (*n* = 6–10 both groups)

We investigated whether exercise alleviated age-related declines in knee joint angles, swelling, stride length, and muscle atrophy. The knee flexion angle and stride length improved in the Ex compared but not in the No-Ex group (Fig. 4A, D, p = 0.006 and 0.001, respectively). The quadriceps fiber CSA significantly increased in mice in the Ex compared to that in the No-Ex group (Fig. 4E, F, p = 0.043). However, the knee extension angle in the Ex group was significantly lower than that in the No-Ex group (Fig. 4B, p = 0.002). No significant difference was observed in the knee widths between the two groups (Fig. 4C).

## Discussion

OA pathogenesis involves not only the breakdown of cartilage but also remodeling of the underlying formation of ectopic bone, hypertrophy of the joint capsule, and inflammation of the synovial lining [26]. The knee joints of SAMP8 showed gradual cartilage destruction, osteophyte formation, and synovitis with age. In addition, the synovium showed an inflammatory response with chemokines that recruit circulating inflammatory cells, such as MCP-1, and the expression of synovial TNF-α, pro-inflammatory macrophages, increased in 9-month-old SAMP8, which might contribute to articular cartilage degeneration. The knee OA changes obtained in our histological and histochemical observations supported to previous findings [19–21]. However, our morphometric observations suggested that SAMP8 developed joint stiffness, and swelling, and walking difficulties associated with knee OA pathologies, which may resemble human OA symptoms. In addition, severe age-related cartilage degeneration was clearly observed in the medial and posterior tibial plateaus. Chondrocyte apoptosis is mainly observed in the medial articular cartilage of the tibial plateaus compared to the lateral side in SAMP8 [21]. The meniscus and cruciate ligament of SAMP8 exhibited pathological changes with cartilage degeneration at 14 weeks of age [21], suggesting that knee joint instability may have occurred in aged SAMP8. Abnormal joint instability contributes to cartilage degeneration and osteophyte formation [27]. Therefore, severe cartilage degeneration and osteophyte formation in SAMP8 may intensify in the medial and posterior regions with increasing age.

This study investigated whether exercise can ameliorate the progression of OA pathologies and symptoms with age in SAMP8. Several studies have reported that moderate treadmill and wheel exercise suppress cartilage degeneration and protect against inflammation in an experimental model of knee OA [28–30]. Our results suggest that histological OA changes in the remaining articular cartilage of anterior tibial plateaus may be alleviated by low-intensity exercise, even when OA is progressing.

The catabolic activity of matrix degradation enzymes such as MMPs is associated with cartilage degeneration and destruction in early OA onset and progression. The over-expression of this enzyme results in progressive degeneration of collagen and aggrecan in the cartilage. The expression of synovial TNF-α has been shown to upregulate the production of proteolytic enzymes such as MMPs, which contribute to articular degeneration [31]. However, although there was no statistical difference, our results showed that the number of MMP-13-positive chondrocytes increased when mice exercised. Histologically, the remaining articular cartilage in the No-Ex group was thin compared to the Ex group, suggesting that cartilage degradation is progressing with age. Therefore, MMP-13-positive chondrocytes in the remaining articular cartilage may be less detectable in the No-Ex group compared that to the Ex group. In fact, the number of MMP13-positive chondrocytes was decreased in 9-month-old mice than that in control mice. In addition, this may be because SAMP8 are genetically predisposed to spontaneously develop knee OA. Therefore, the expression of MMP-13 in the remaining cartilage may be associated with cartilage degeneration associated with aging, even when cartilage degradation is alleviated through exercise. Hubbard-Turner et al. [32] examined the effects of lifelong physical activity in spontaneous developed knee OA using C57Bl/J mice. They reported that physical activity protects the joint from degeneration during the first 12 months of life, but, thereafter, there was no protective benefit. C57Bl/J mice are genetically predisposed to develop knee OA at 9 months of age [32]. Taken together, their study suggested that physical activity may have temporarily protected C57Bl/J mice from the progression of early-onset OA, but physical activity may have no impact on the genetic predisposition C57Bl/J mice. Thus, As in C57Bl/J mice, exercise may similarly have no impact on genetic predispositions in SAMP8. Therefore, increased MMP-13 expression in the remaining articular cartilage of the Ex group may be indicative of impending cartilage degeneration.

Our results suggested that exercise might promote cartilage degeneration in the medial and posterior knee regions in SAMP8. However, moderate physical activity reduced levels of pro-inflammatory molecules (TNF-α) and increased levels of a chondroprotective marker (lubricin) in the synovium of the experimental OA model rats [10]. In addition, physical activity promotes lubricin synthesis in the articular cartilage, thereby preventing cartilage degradation in elderly rats [33]. Our results showed that exercise increased collagen type II in the remaining cartilage and suppressed synovial inflammation by reducing pro-inflammatory macrophage infiltration. Although the expression of MMP-13 in the cartilage was not changed through exercise, this therapy may have decreased levels of pro-inflammatory cytokines in the synovium and temporarily inhibited inflammatory factor-mediated cartilage degradation in SAMP8. However, knee swelling did not change statistically following exercise intervention. Therefore, the relationship between decreased synovitis and knee swelling may not have been detected by our method.

This study suggested that low-intensity exercise improved OA symptoms such as age-related declines in knee flexion angle, stride length, and muscle atrophy. SAMP8 previously displayed an age-related decline in locomotor activity from approximately 7 months of age [34]. The alleviated remaining cartilage degeneration and synovitis may contribute to improving these joint functions and walking ability. In contrast, atrophy of the quadriceps muscles triggers cartilage degeneration of the knee OA model animals [35]. Furthermore, treadmill running is beneficial for muscle hypertrophy in SAMP [36]. Therefore, preventing knee joint motion and quadriceps muscle atrophy through exercise may attenuate cartilage degeneration and synovitis in SAMP8. However, the knee extension angle was not improved through exercise, suggesting that this exercise program may not have been adequate to improve reduced knee extension with aging. Therefore, the decline in knee joint range of motion due to OA with aging may require additional rehabilitation programs, such as manual therapy. Our histological observations suggest that aged SAMP8 may have knee joint instability. Although exercise alleviated the cartilage degeneration of the remaining cartilage and synovitis, joint instability may have promoted cartilage degeneration and decreased joint motion such as knee extension. Oka et al. [8] reported that exercise with control of abnormal joint movements reduced synovitis, which suppressed cartilage degeneration in an experimental OA model. When applying this principle to older people with OA, combined programs of walking exercises and knee stabilization training, such as muscle strength exercises, may be beneficial in preventing the progression of OA with age.

Macrophage infiltration in the synovium is common in OA [37]. The pro-inflammatory macrophage contributes to OA, whereas the anti-inflammatory macrophage can reverse it or favor the chondrogenic process [38]. Pro-inflammatory cytokines induced synovial inflammation [37], whereas physical exercise reduced pro-inflammatory molecules and increased anti-inflammatory cytokines [10]. Our results showed that exercise suppressed synovial inflammation by reducing pro-inflammatory macrophage infiltration and may contribute to reducing the degeneration of the remaining articular cartilage during OA progression. The synovium displays a superficial cellular lining that is composed of two types of synoviocytes: type A cells containing vacuoles that are related to phagocytic functions (macrophages) and type B cells with secretory fibroblast-like functions producing hyaluronan and lubricin [10]. Therefore, TNF-α may be produced by type A cells, and exercise during OA progression reduced its expression in SAMP8. In addition, exercise has been shown to have systemic anti-inflammatory effects in healthy older individuals and several patients with disease, such as those suffering from coronary artery disease and cancer survivors [39]. Therefore, low-intensity exercise may be a therapeutic strategy to ameliorate degeneration of the remaining articular cartilage and synovitis by reducing pro-inflammatory macrophage infiltration.

Our study had some limitations. First, this study examined mice through histological and immunohistochemical analyses only. Further studies are required to investigate the physiological and pathological changes in the cartilage, synovium, and synovial fluid. In the immunohistochemical analyses, collagen type II immunostaining in cartilage matrix may be affected by decalcification solution. In addition, the semi-quantitative analysis in the collagen type II and MMP-13 immunostainings calculated the number of positive cells per unit area. This study compared between the groups using the number of immune-positive cells per unit area. However, the chondrocytes undergo apoptosis and other forms of cell death during OA progression. Therefore, further analysis may be required including the calculation of the percentage of immune-positive cells relative to the total number of cells in the cartilage. Second, it is still unclear which exercise regimen is beneficial and what the optimal dose (phase of intervention, exercise type, intensity, and frequency) of exercise is that can ameliorate OA. Third, we used a small sample size for each month to minimize the number of animals used. Forth, we examined only male animals. Clinically, females are at higher risk of OA in humans. Further studies are required to clarify the differences between male and female animals. Despite these limitations, this study suggests that SAMP8 prematurely develop spontaneous knee OA, which is associated with a decline in knee flexion angle, knee swelling, and stride length. In addition, low intensity exercise may temporarily alleviate the degeneration of remaining cartilage and synovitis in SAMP8, even if exercise was performed during OA progression.

## Conclusion

This study demonstrated that male SAMP8 developed spontaneous knee OA that resembled human OA. Exercise therapy during the progression of OA may promote cartilage degeneration in the medial and posterior regions in SAMP8. However, this study suggested that exercise may suppress the degeneration of remaining cartilage and synovitis through reduced synovial inflammation. Furthermore, exercise therapy improved age-related decreases in knee flexion angle, stride length, and quadriceps muscle atrophy in SAMP8. However, SAMP8 are genetically predisposed to develop spontaneous knee OA, and cartilage degeneration will inevitably progress with age. Therefore, the present study suggests that exercise may be provide temporarily alleviation of cartilage degeneration and synovitis in SAMP8. Exercise during the progression of OA with age may be coupled to mechanical stress that could be both beneficial and detrimental to joint health.

### Supplementary Information

**Additional file 1:** **Supplementary Figure 1.** Comparison of morphometric assessments between 3- and 9-month-old SAMP8. The acute angle long axis of the femur and fibula formed by three points of the femoral greater trochanter, center of patella, and lateral malleolus were measured as knee flexion (A, B) and extension (C, D) angles. Both sides’ hindlimbs were measured for the maximal width in the central part of the knee (E, F).

**Additional file 2:** **Supplementary Figure 2.** Macroscopic observation of the knee joint. Macroscopic observation of the joint capsule (A) and cartilage surface (B) in 8- or 9-month-old SAMP8 and control mice. The medial joint capsule in SAMP8 was thicker than that in the control mice (*).M: medial side, L: lateral side. (*n* = 4–5 per age group).

**Additional file 3:** **Supplementary Figure 3.** Articular cartilage alteration and synovial inflammation response with aging by immunohistochemical analysis. A: Immunoreactivity of collagen type II and MMP-13 positive chondrocytes in the remaining cartilage of the tibial plateau and MCP-1- and TNF-α-positive cells in the posterior synovium. Photomicrographs of each immunostaining were obtained from the right rectangular area of Fig. 1A. High magnification panels show immune-positive cells (black arrow head) and immune-negative cells (white arrow head) of collagen type II- and MMP-13-positive chondrocytes. B: The number of collagen type II-positive chondrocytes. C: The number of MMP-13-positive chondrocytes. D: The percentage of MCP-1-positive cell areas. E: The percentage of TNF-α-positive cell areas. The data from 9-month-old SAMR1 was used as a control. Data are expressed as mean ± 95% CI. *p < 0.05, **p < 0.01, ***p < 0.001. Scale bars = 50 μm (all panels) and 25μm (all high magnification panels) (*n* = 6 per age group).

## Data Availability

The data on which this study is based is available from the corresponding author upon reasonable request.

## Abbreviations

OA: Osteoarthritis
SAMP8: Senescence-accelerated mouse prone 8
SAMR1: Senescence-accelerated mouse resistant 1
OARSI: Osteoarthritis research society international
ACL-T: Anterior cruciate ligament transection
CSA: Cross-sectional area
MMP: Matrix metalloproteinase
MCP: Monocyte chemoattractant protein
TNF: Tumor necrosis factor
