# Supplementary figures and images for: Effects of low-intensity exercise on spontaneously developed knee osteoarthritis in male senescence-accelerated mouse prone 8
Source: Arthritis Res Ther. 2023 Sep 14;25:168. doi: 10.1186/s13075-023-03162-z (PMC10500802; doi:10.1186/s13075-023-03162-z)

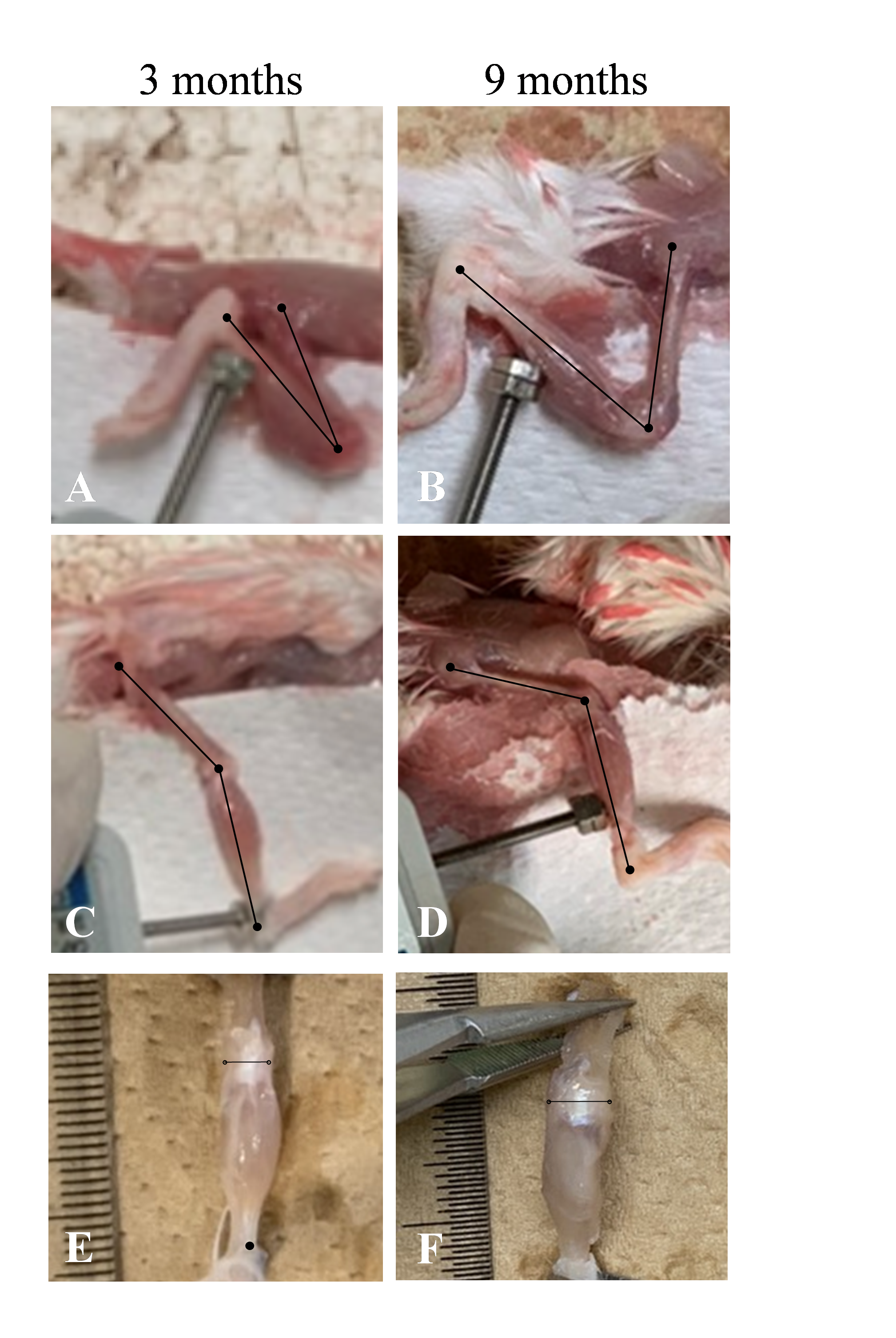

Supplement: Supplementary file 1 — Additional file 1: Supplementary Figure 1. Comparison of morphometric assessments between 3- and 9-month-old SAMP8. The acute angle long axis of the femur and fibula formed by three points of the femoral greater trochanter, center of patella, and lateral malleolus were measured as knee flexion (A, B) and extension (C, D) angles. Both sides’ hindlimbs were measured for the maximal width in the central part of the knee (E, F). [file 13075_2023_3162_MOESM1_ESM.tif]

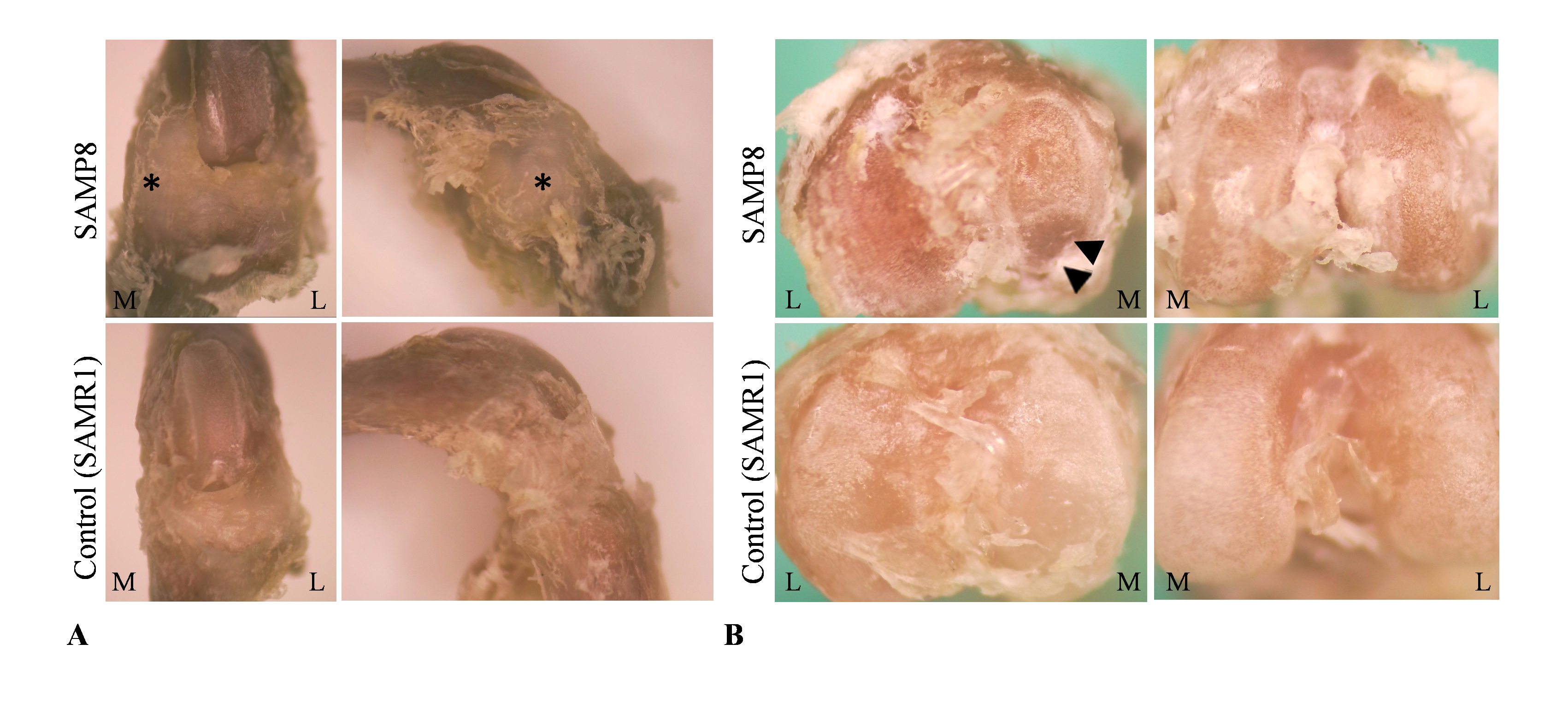

Supplement: Supplementary file 2 — Additional file 2: Supplementary Figure 2. Macroscopic observation of the knee joint. Macroscopic observation of the joint capsule (A) and cartilage surface (B) in 8- or 9-month-old SAMP8 and control mice. The medial joint capsule in SAMP8 was thicker than that in the control mice (*).M: medial side, L: lateral side. (n = 4–5 per age group). [file 13075_2023_3162_MOESM2_ESM.tif]

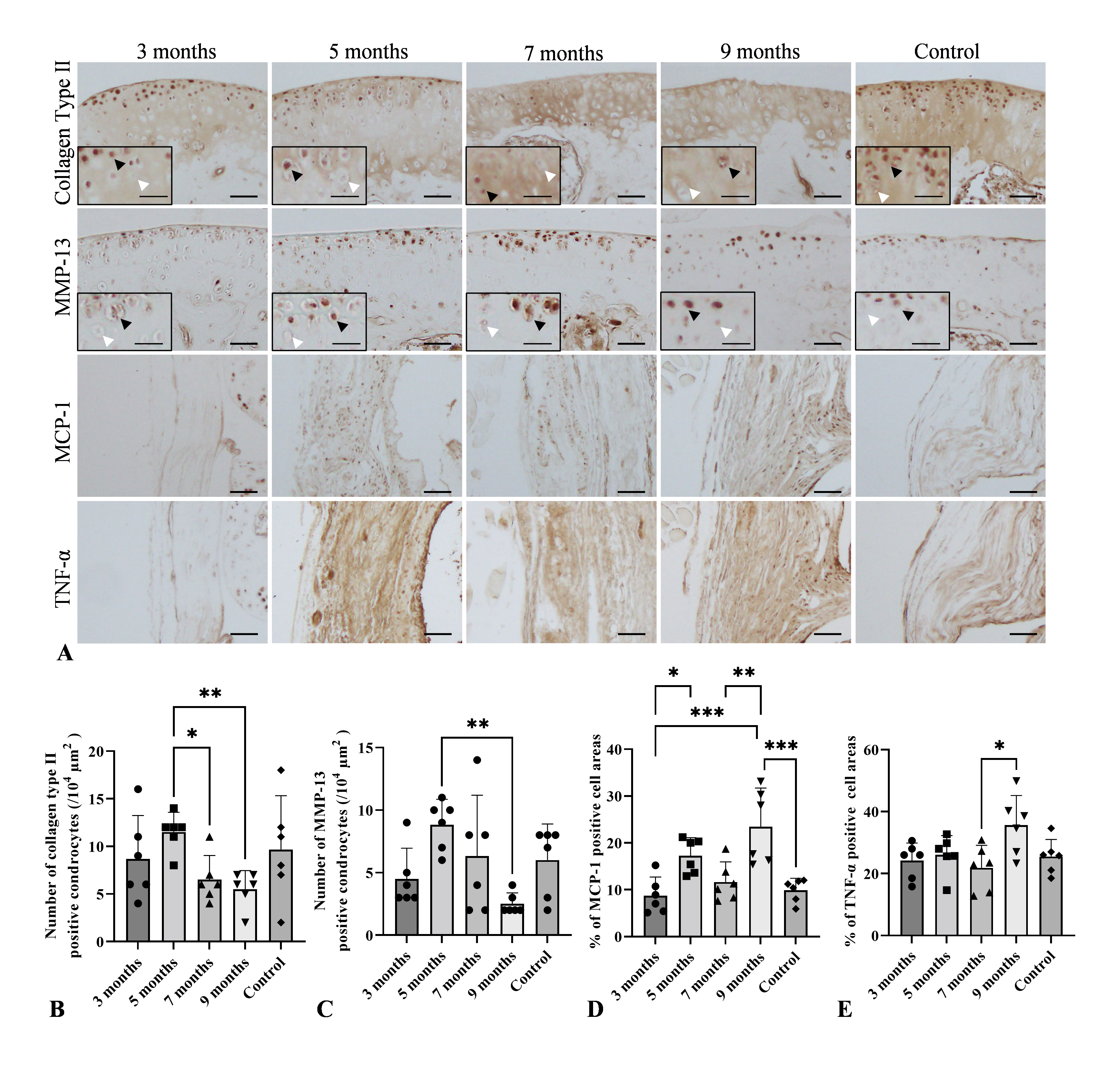

Supplement: Supplementary file 3 — Additional file 3: Supplementary Figure 3. Articular cartilage alteration and synovial inflammation response with aging by immunohistochemical analysis. A: Immunoreactivity of collagen type II and MMP-13 positive chondrocytes in the remaining cartilage of the tibial plateau and MCP-1- and TNF-α-positive cells in the posterior synovium. Photomicrographs of each immunostaining were obtained from the right rectangular area of Fig. 1A. High magnification panels show immune-positive cells (black arrow head) and immune-negative cells (white arrow head) of collagen type II- and MMP-13-positive chondrocytes. B: The number of collagen type II-positive chondrocytes. C: The number of MMP-13-positive chondrocytes. D: The percentage of MCP-1-positive cell areas. E: The percentage of TNF-α-positive cell areas. The data from 9-month-old SAMR1 was used as a control. Data are expressed as mean ± 95% CI. *p < 0.05, **p < 0.01, ***p < 0.001. Scale bars = 50 μm (all panels) and 25μm (all high magnification panels) (n = 6 per age group). [file 13075_2023_3162_MOESM3_ESM.tif]
